# Genome-scale data reveal the role of hybridization in lichen-forming fungi

**DOI:** 10.1038/s41598-020-58279-x

**Published:** 2020-01-30

**Authors:** Rachel Keuler, Alexis Garretson, Theresa Saunders, Robert J. Erickson, Nathan St. Andre, Felix Grewe, Hayden Smith, H. Thorsten Lumbsch, Jen-Pan Huang, Larry L. St. Clair, Steven D. Leavitt

**Affiliations:** 1 Department of Biology, Brigham Young University, 4102 Life Science Building, Provo, UT 84602 USA; 2 Grainger Bioinformatics Center, Science & Education, The Field Museum of Natural History, 1400 S. Lake Shore Drive, Chicago, IL 60605 USA; 3 Biodiversity Research Center, Academia Sinica, 128 Academia Rd, Section 2, Nankang District, Taipei, 11529 Taiwan; 4 M. L. Bean Life Science Museum, Brigham Young University, 1115 MLBM, Provo, UT 84602 USA

**Keywords:** Fungal evolution, Fungal genomics, Fungi, Evolution, Phylogenetics, Speciation, Taxonomy

## Abstract

Advancements in molecular genetics have revealed that hybridization may be common among plants, animals, and fungi, playing a role in evolutionary dynamics and speciation. While hybridization has been well-documented in pathogenic fungi, the effects of these processes on speciation in fungal lineages with different life histories and ecological niches are largely unexplored. Here we investigated the potential influence of hybridization on the emergence of morphologically and reproductively distinct asexual lichens. We focused on vagrant forms (growing obligately unattached to substrates) within a clade of rock-dwelling, sexually reproducing species in the *Rhizoplaca melanophthalma* (Lecanoraceae, Ascomycota) species complex. We used phylogenomic data from both mitochondrial and nuclear genomes to infer evolutionary relationships and potential patterns of introgression. We observed multiple instances of discordance between the mitochondrial and nuclear trees, including the clade comprising the asexual vagrant species *R. arbuscula*, *R. haydenii*, *R. idahoensis*, and a closely related rock-dwelling lineage. Despite well-supported phylogenies, we recovered strong evidence of a reticulated evolutionary history using a network approach that incorporates both incomplete lineage sorting and hybridization. These data suggest that the rock-dwelling western North American subalpine endemic *R. shushanii* is potentially the result of a hybrid speciation event, and introgression may have also played a role in other taxa, including vagrant species *R. arbuscula*, *R. haydenii* and *R. idahoensis*. We discuss the potential roles of hybridization in terms of generating asexuality and novel morphological traits in lichens. Furthermore, our results highlight the need for additional study of reticulate phylogenies when investigating species boundaries and evolutionary history, even in cases with well-supported topologies inferred from genome-scale data.

## Introduction

While once considered an evolutionary dead end, hybridization is more pervasive than previously assumed, found among plants, fungi, and animals^1–6^. Neglecting to consider the potential influence of hybridization on speciation and delimiting species boundaries risks oversimplifying evolutionary inferences or may lead to erroneous or conflicting interpretations. Both the extent of the impact of hybridization on speciation and the ability to detect it are highly debated in the scientific community^7–10^; nevertheless, there remains value in exploring the influences of interspecific gene flow on species as a whole.

Hybridization can play a role in evolutionary dynamics and the transmittance of adaptive variation^11^, potentially leading to adaptation to new niches or habitats^12^ and the introduction of novel phenotypes^13^. Examples include the hybrid exchange of mimicry loci among *Heliconius* butterfly species^14^, transgressive segregation of Darwin’s finches (*Geospiza* spp.)^15^, and introgressive hybridization enabling the exchange of flower color mutations in monkeyflowers (*Mimulus* spp.)^16^. Genetic interactions can result in incompatibilities between parental alleles, referred to as Dobzhansky-Muller incompatibilities, which can negatively affect fitness or cause hybrid sterility^17,18^. Additionally, hybridization can manifest in the genome as a discordance between mitochondrial and nuclear markers, which in turn can influence the genome in both adaptive and maladaptive ways^5,19–22^. Adaptive introgression of mitochondrial DNA may play an important role in speciation and phylogeography^23,24^.

While hybridization has been well-documented among pathogenic fungi^21,25–30^, the role of hybridization on the process of speciation of fungal lineages with different life histories and ecological functions is not well understood, including among lichen-forming fungi. The role of gene flow and hybridization in lichen-forming fungal evolution has been a long-standing question^31–35^. Species boundaries in fungi, including symbiotic fungi such as lichen formers, have received substantial attention and become more robust with molecular sequence data^36^. However, processes involved in speciation in lichen-forming fungi have received far less attention. With a poor fossil record^37,38^ and potentially unreliable phenotype-based classification^39^, options for elucidating the evolutionary history of lichen-forming fungi was challenging prior to the development of modern molecular tools. Potential evidence of hybridization has been encountered in a number of lichen-forming fungal lineages^33,40–43^. However, genome-scale datasets have not yet been used to explicitly infer the role of hybridization on the process of speciation lichen-forming fungi.

Characterizing gene flow is further complicated by a diverse range of reproductive strategies used by lichen-forming fungi^44,45^. Species of filamentous fungi with one of two different allelic variants can obligately cross-fertilize (heterothallism), only being able to reproduce with an individual with the opposite allelic variant, in contrast to species which contain both allelic variants and can self-fertilize (homothallism)^46–48^. Asexual reproduction is also common in many fungi^47^, including lichens that commonly reproduce asexually via vegetative fragmentation or propagules^44^. Hybridization may occur among interfertile species via sexual reproduction^25^, and while not yet explored in lichen-forming fungi, plant pathogenic fungi have been shown to hybridize asexually via fusion of hyphae^3,49^.

**Figure 1:**
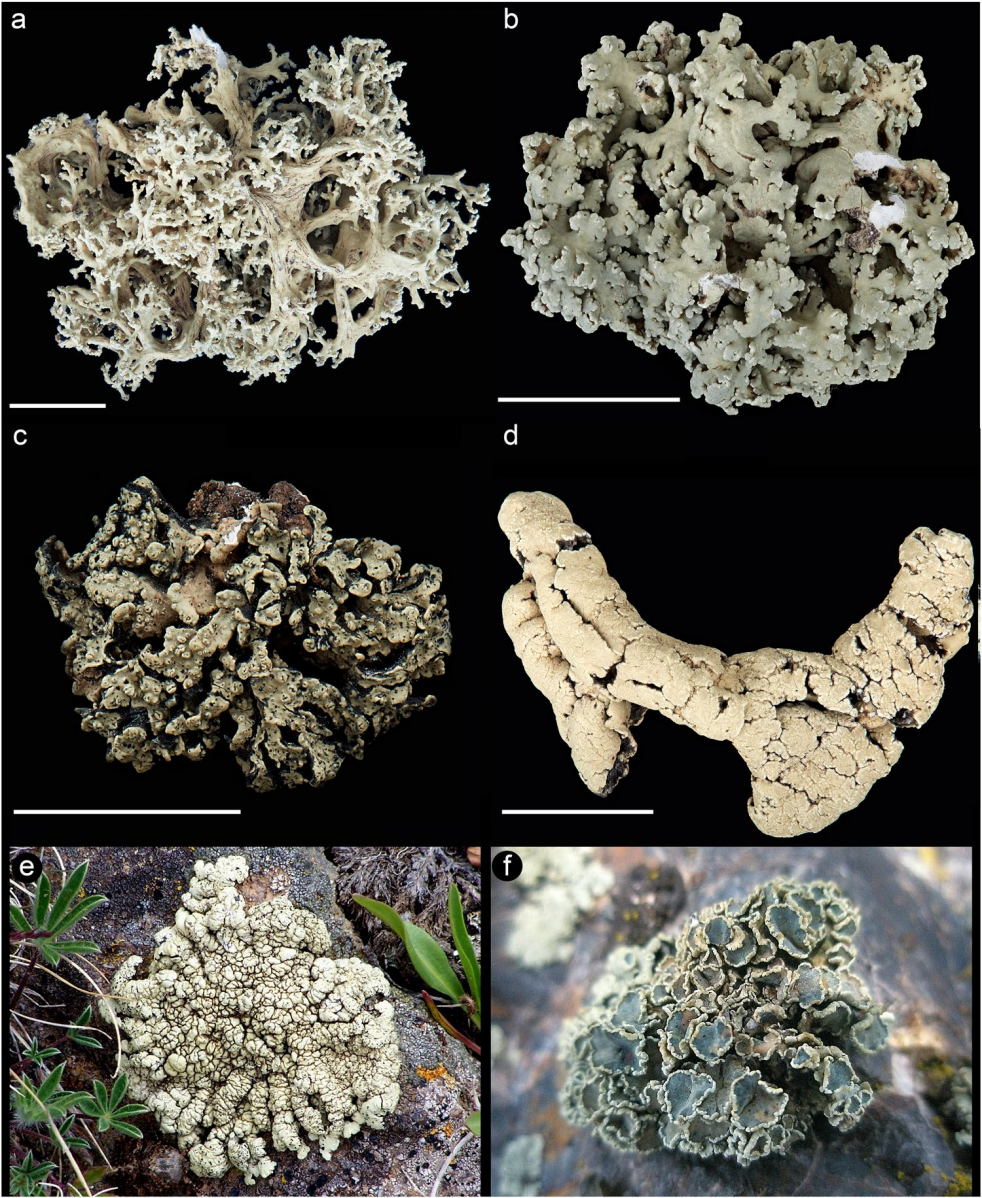
Morphological variation in the *Rhizoplaca melanophthalma* complex. Size bar = 1 cm. (**a**) Vagrant taxon *Rhizoplaca arbuscula*; (**b**) vagrant taxon *Rhizoplaca haydenii*; (**c**) a member of the ‘saxicolous *haydenii* population’, a putative hybrid population (*Leavitt 715* [BRY-C]); (**d**) vagrant taxon *Rhizoplaca idahoensis*; (**e**) saxicolous *Rhizoplaca shushanii*, a taxon inferred here to be of hybrid origin—field image; and (**f**) a member of the saxicolous *Rhizoplaca porteri* group with abundant fruiting bodies (apothecia)—field image.

To better understand the potential role of hybridization in the diversification processes in symbiotic fungi, we investigated a morphologically diverse clade of lichen-forming fungi with a Pleistocene-dominated diversification history, the *Rhizoplaca melanophthalma* complex (Fig. 1)^50^. While species boundaries within this complex have been well-studied^51^, processes that drive diversification are poorly understood. Members of this species complex are distributed worldwide, with rock-dwelling (saxicolous) species common in montane regions, hot deserts, and cold deserts, including Antarctica. In many regions, distinct species occur in sympatry with no evidence of ongoing or recent gene flow^52^. Vagrant forms (Fig. 1a–d)—which grow obligately unattached to substrates, therefore mobile with wind or water movement—occur only in the cold steppe regions of Western North America^53^. While the rock-dwelling species are abundantly fertile, vagrant forms rarely produce fruiting bodies (apothecia), reproducing instead by vegetative fragmentation. However, in some cases environmentally modified erratic forms may become detached from rocks (facultatively unattached) and continue living on the soil with apothecia. At some sites in the Intermountain Western region of North America, attached, erratic, and vagrant forms of *Rhizoplaca* occur in sympatry^53^.

These striking differences in growth form (attached, relatively small vs. large, unattached, unusual morphologies) and reproductive strategies (sexual vs. strictly asexual) among closely related species-level lineages (Fig. 1) beg the question of which evolutionary processes drive this disparity. Furthermore, considering the frequently sympatric distributions and recent diversification history^53^, the origin and establishment of reproductive barriers in the *R. melanophthalma* complex remain unknown. Given the known role of hybridization in developing novel traits and reproductive barriers in fungi^21,25,54^, here we investigate whether hybridization played a role in the process of diversification and establishment of reproductive barriers among members in the *R. melanophthalma* complex.

To address this question, we targeted vagrant *Rhizoplaca* forms in sage-steppe communities in western North America. While previous phylogenomic studies have provided well-supported hypotheses of evolutionary relationships with the *Rhizoplaca melanophthalma* species complex^51,55,56^, the evolutionary origin of vagrancy remains unresolved. Vagrant taxa include *Rhizoplaca arbuscula* Rosentr., St. Clair & Leavitt, *R. haydenii* (Tuck.) W.A. Weber, *R. idahoensis* Rosentreter & McCune, and two subspecies of *R. melanophthalma*, *R. melanophthalma* subsp. *cerebriforms* Rosentreter & B.D. Ryan and *R. melanophthalma* subsp. *crispa* Rosentreter & B.D. Ryan. *Rhizoplaca arbuscula* was recently elevated from subspecies, *R. haydenii* subsp. *arbuscula*, to species based on phylogenomic data and distinct phenotypic characters^57^. *Rhizoplaca arbuscula*, *R. haydenii*, and *R. idahoensis* are distinct from other species in the complex because they are exclusively asexual, obligately unattached (true vagrants), and morphologically distinct. Previous studies revealed potential discordance at a genomic level^55,58^, and we hypothesize that hybridization may have played a crucial role in the origin of these deviant lineages. To do this, we used genome-scale data to (1) reconstruct evolutionary relationships, (2) identify potential cases of mtDNA introgression, and (3) infer potential hybridization events to examine patterns of reticulation in the *R. melanophthalma* group.

## Methods

### Taxon sampling

Metagenomic data was generated from 55 specimens representing the nine formally described species within the *Rhizoplaca melanophthalma* species complex (Supplementary Table S1; short reads generated for this study were deposited in NCBI’s Short Read Archive under project PRJNA576709)^52^, as well as representative specimens of three formally described subspecies^59^. Previous studies have provided strong support for some species boundaries and relationships among species-level lineages in this complex^51,56,58,60^, with the exception of members of the *R. porteri* group and the vagrant taxa/forms, including: *R. arbuscula*, *R. haydenii*, *R. idahoensis*, *R. melanophthalma* subsp. *cerebriformis*, and *R. melanophthalma* subsp. *crispa* (Fig. 1). Vagrant specimens were sampled from multiple sites in the Lemhi Valley and Big Lost River Valley in central Idaho where distinct vagrant taxa/forms commonly co-occur^59^, supplemented by a limited number of specimens collected at multiple sites in Wyoming, USA (Supplementary Table S1). *Rhizoplaca arbuscula* and *R. melanophthalma* subsp. *cerebriformis* were represented by material collected from the type localities. We also included a saxicolous specimen collected in western Montana that had been recovered as closely related to *R. haydenii* in a previous, multi-locus phylogenetic study^60^, provisionally called ‘saxicolous *haydenii* 715f’. Based on evidence suggesting potential mtDNA introgression in this specimen, multi-locus sequence data was generated for eight additional morphologically similar thalli collected from the same location in western Montana (Supplementary Fig. S1), hereafter called the ‘saxicolous *haydenii* population’ (Fig. 1c), in hopes of identifying additional specimens of putative hybrid origin. *Rhizoplaca novomexicana* was used as our outgroup^55^.

### DNA extraction and sequencing

For the 25 new specimens collected for metagenomic high-throughput sequencing, total genomic DNA was extracted from a small portion of lichen thalli (comprised of the mycobiont, photobiont, and other associated microbes) using the E.Z.N.A. Plant DNA DS Mini Kit (Omega Bio-Tek, Inc., Norcross, GA, USA) following the manufacturers’ recommendations. Total genomic DNA was prepared following the standard Illumina whole genome sequencing library preparation process using Adaptive Focused Acoustics for shearing (Covaris); followed by an AMPure cleanup step. The DNA was then processed with the NEBNext Ultra™ II End Repair/dA-Tailing Module end-repair and the NEBNext Ultra™ II Ligation Module (New England Biolabs) while using standard Illumina index primers. Libraries were pooled and sequenced with the HiSeq. 2500 sequencer in high output mode at the DNA Sequencing Center, Brigham Young University, Provo, Utah, USA, using 250 cycle paired-end reads, 300 cycle paired-end reads, or 50 cycle single-reads (six samples).

For the eight specimens of putative hybrid origin from the ‘saxicolous *haydenii* population’ (Supplementary Fig. S1), DNA was extracted using the Wizard Genomic DNA Purification Kit (Promega). Since the entire genomes of these specimens had not been sequenced with the first 25 specimens, we sequenced two nuclear markers using Sanger sequencing, the internal transcribed spacer region (ITS)^61^ and a fragment of the HEC/Ndc80p family protein, along with a region of the mitochondrial genome. The ITS marker was amplified with primers previously published^56^. For the HEC/Ndc80p marker, we used primers ‘rhizoHEC-f’ (5′-CTTCGGTTTCTCTCTCGGCA-3′) with ‘rhizoHEC-r’ (5′-ACCTCGCCGACACAAAAAGA-3′). The mitochondrial marker, a variable region 720 to 965 bp, was amplified with custom-designed primers for this group; i.e. ‘rhizoMTsp-f’ (5′-GCCCAYGGGTTTGTTTCTTC-3′) with ‘rhizoMTsp-r’ (5′-TGGCCGAGGAGGACTATTGA-3′). Markers were selected based on exploratory analyses of previous genome assemblies^55^ to identify a single-copy nuclear marker and a mitochondrial marker that consistently recovered *Rhizoplaca* species as monophyletic. We were unable to identify a single genomic region that recovered members of the *R. porteri* group (Fig. 1f; *R. occulta, R. polymorpha*, and *R. porteri*) as monophyletic, except for the ITS region. PCR cycling conditions for all markers were: initial denaturation at 95 °C for 10 min, followed by 38 cycles at 94 °C for 1 min, 50 °C for 1 min, 72 °C for 2 min, and final elongation at 72 °C for 10 min. PCR fragments were cleaned using the ExoSAP-IT PCR Product Cleanup Reagent (ThermoFisher Scientific), and complementary strands were sequenced using the same primers used for amplification. Sequencing reactions were performed using Big Dye 3.1 (Applied Biosystems, Foster City, CA), and products were run on an AB 3730xl automated sequencer at the DNA Sequencing Center, Brigham Young University, Provo, UT, USA.

### Read filtering and phylogenomic data matrices

All reads were filtered using TRIMMOMATIC v0.33 before assembly to remove adapter and primer sequences, low quality reads, and/or included contamination from Illumina adaptors. Reads were trimmed when the average quality of 5-base sliding windows was below 20 and bases at the start and end of reads had a quality below 3 and 10, respectively. Subsequently, all trimmed reads shorter than 36 bp were filtered out^61^.

To infer evolutionary relationships and assess potential incongruences between the nuclear and mitochondrial genomes, we generated genome-scale datasets for both genomes. For the nuclear genome, we generated (i) a genome-scale alignment, (ii) a SNP dataset derived from variable sites in the Benchmarking Universal Single-Copy Orthologs (BUSCO)^62^, and (iii) gene trees from 407 single-copy core eukaryotic genes. Because phylogenomic datasets were generated from short-read, metagenomic data (reads from lichen symbionts and associated organisms), exploratory phylogenetic analyses, BLAST comparisons, and relative sequencing coverage were assessed to ensure the origin from the mycobiont nuclear and mitochondrial genomes. Datasets and accompanying analyses are summarized in Table 1.

**Table 1:** Summary of phylogenomic datasets and associated analyses.

Dataset	Alignment length	Missing data	Analyses
nuclear REALPHY	18,457,947	6.0%	Concatenated ML (IQ-TREE)
mtDNA REALPHY	63,877	12.4%	Concatenated ML (IQ-TREE)
BUSCO 1 kb + SNPs	142,437	7.0%	Species tree inferred using quartet amalgamation (SVDquartets) & ABBA/BABA test of introgression (HyDe)
407 CEG trees	NA	0%	Summary species tree (ASTRAL-III)/network approach incorporating both incomplete lineage sorting and hybridization (MPL, PhyloNet)

The nuclear REALPHY dataset was generated using contigs >5 kb from a draft genome assembly of *R. melanophthalma* as the reference; the mitochondrial REALPHY dataset was generated using the longest assembled mitochondrial contig; the ‘BUSCO 1 kb + SNPs’ dataset represents all polymorphic sites from concatenated alignments of all BUSCO gene regions >1 kb; and the ‘407 CEG trees’ are gene topologies inferred from 407 core eukaryotic gene regions passing quality filters. Alignment lengths and percentage of missing data are given for each alignment.

The nuclear phylogenomic alignment was created with REALPHY v1.12^63^, which has previously been shown to construct robust genome-scale datasets^55,64^. Metagenomic reads were mapped to contigs >5 kb from a previously published draft genome assembly for *R. melanophthalma sensu stricto*^55^ in REALPHY v1.12, implementing Bowtie 2.1.0^65^ for read mapping using the following parameters: -readLength 75 –perBaseCov 5 –gapThreshold 0.2 with the remaining parameters set at default values. With the –gapThreshold parameter set to 0.2, each site had no more than 20% missing data.

For the nuclear SNP dataset, the fungal set of BUSCO regions were extracted from the draft genome assembly for *R. melanophthalma s. str*.^55^. BUSCO uses reciprocal best hit, creating a Hidden Markov Model (HMM) profile using the protein sequences of 50 reference genomes for each single-copy gene. Every HMM profile generated was then used as query in tBLASTn searches against each genome to find the putative genomic region. An AUGUSTUS^66^ prediction was performed for each of the genomic regions. Predicted genes were then aligned to the HMM profiles of the BUSCO gene, and only the gene with the highest bit-score was kept. The BUSCO analysis was conducted in the Cyverse.org Discovery Environment^67,68^. Single-copy BUSCO gene regions predicted in the *R. melanophthalma* reference genome were extracted; and duplicated genes and fragments less than 1 kb were removed. A supermatrix was assembled by concatenating every BUSCO single-copy gene using FASconCAT.pl^69^. Metagenomic reads were mapped to BUSCO genes larger than 1 kb using REALPHY v1.12, implementing the same parameters as above, and variable sites were extracted for a ‘SNP’ dataset. We also generated gene trees for a subset of the BUSCO genes, focusing on exon regions of 458 core eukaryotic genes (CEG) and extracted using CEGMA^70^. We used the ‘map_n_extract’ pipeline (https://github.com/felixgrewe/map_n_extract/^55^) to map metagenomic reads from each specimen to CEG genes predicted in the *R. melanophthalma* reference genome. Resulting consensus sequences from each sample were filtered by removing insertions not present in the reference CEG regions and only including genes in which the reference starts with ATC and ends with TGA, TAA, or TAG. We excluded CEG genes with a stop codon within the coding region or those with coding regions that were not divisible by three. Consensus sequences for each CEG region, including introns and small portions of upstream and downstream regions, were aligned using the program MUSCLE^71^. Exons from individual CEG consensus sequences passing filtering parameters were extracted for phylogenetic analysis.

For the mitochondrial genome dataset, metagenomic reads from each library were assembled using the SPAdes v3.12.0 assembler^72^. We selected the longest mitochondrial assembly as a reference for phylogenomic assembly using REALPHY v1.12. The following parameters were implemented in REALPHY, implementing Bowtie v2.1.0^65^ for read mapping: -readLength 100 -perBaseCov 5 -gapThreshold 0.2, with the remaining parameters set at default values. For exploratory comparisons, we generated additional mitochondrial datasets, inferring phylogenies from (i) concatenated CDS (protein coding sequence) regions, (ii) concatenated intronic regions, and (iii) complete, aligned mitochondrial contigs. We used DOGMA^73^ to initially demark mitochondrial genomic features. Multiple sequence alignments for individual CDSs and introns were performed using MAFFT^74^, with the resulting CDS and intron alignments concatenated separately and implementing the G-INS-I option. Complete mitochondrial contigs were also aligned using MAFFT, implementing the E-INS-I option.

### Species tree inference from phylogenetic data

Evolutionary relationships were inferred using (i) a supermatrix approach^75^, (ii) two computationally efficient species tree inference methodologies which accounted for incomplete lineage sorting, using ASTRAL-III^76^ and SVDQuartets^77^, and (iii) a recently developed network approach that incorporates both incomplete lineage sorting and hybridization implemented in PhyloNet^78^. In some cases, concatenation, or supermatrix approaches, have been shown to accurately infer relationships across a wide range of scenarios^75^. We inferred relationships from both the nuclear and mitochondrial alignments generated using REALPHY. Phylogenetic trees were reconstructed using maximum likelihood as implemented in IQ-TREE (Version 1.6.7)^79^, with 1,000 ultra‐fast bootstrap replicates^80^ to assess nodal support, followed by the best‐fit substitution model as predicted by ModelFinder^81^. Topologies inferred from the nuclear and mitochondrial datasets were visually assessed to infer cases of mitochondrial introgression with *a posteriori* comparisons of morphological groups with phylogenetic structure.

Because standard concatenation approaches may return incorrect trees with high support in the presence of incomplete lineage sorting^82^, we used two “coalescent-based” methods to infer species trees for the *R. melanophthalma* complex: ASTRAL-III^77^, a summary method which accepts gene trees as input to generate a species tree, and SVDQuartets^78^, as implemented in PAUP*, a method that infers relationships among quartets of taxa under the coalescent model. Both ASTRAL-III and SVDQuartets + PAUP* are computationally efficient and are able to accurately infer relationships under a range of scenarios^83^. Specimens were assigned to species/populations based on previous studies and candidate species-level lineages delimited here. A total of nine species/populations were defined for species tree inference. Based on our analyses, *R. melanophthalma, R. parilis*, and *R. shushanii* are robustly delimited^55^. Previous studies have highlighted the close relationship between *R. occulata*, *R. polymorpha*, and *R. porteri*^58^. Our sampling was not designed to delimit species boundaries or relationships among these three taxa; therefore, we treated them as a single species/population—the *R. porteri* group. Species boundaries in the vagrant taxa, e.g., *R. arbuscula*, *R. haydenii,* and *R. idahoensis sensu lato*, were delimited using an iterative approach comparing morphology with the mitochondrial phylogeny. Four mitochondrial clades were identified that correlated with distinct morphological groups (see Results), and these four clades were treated as distinct species/populations in the species tree reconstructions using nuclear phylogenomic data. The single specimen with clear mitonuclear incongruence, specimen ‘715f’ from western Montana (Fig. 1c), was treated as a distinct species in all species tree analyses.

We used ASTRAL-III v5.6.3 to generate a summary species tree based on the 407 generated CEG gene trees that passed quality filtering. ASTRAL-III reconstructs species trees using the minimum quartet distance. Branch support values were inferred using local posterior probabilities^84^. SVDquartets^77^, as implemented in PAUP* v.4.0a^85^, were used to generate a species tree from SNP data obtained from the BUSCO alignment generated by REALPHY. This approach accounts for incomplete lineage sorting (ILS) without the need to assemble gene trees. Support for the final species tree was assessed using 25,000 random quartets and n = 1000 bootstrap replicates.

Incongruence among individual gene topologies based on the 407 CEG gene topologies was evaluated using internode certainty (IC) and relative tree certainty (TCA) metrics^86^ as implemented by RAxML v8.2.3. The IC value of a given internode reflects its specific degree of incongruence, while the TCA value characterizes the global degree of incongruence among trees.

### Tests for reticulated relationships

Mitonuclear incongruence suggested a hybrid origin for members of the ‘vagrant clade’ clade, comprising *R. arbuscula*, *R. haydenii*, *R. idahoensis*, and the ‘saxicolous *haydenii* population’ (see Results). We then used maximum pseudolikelihood (MPL) to assess corroborative evidence supporting this conclusion. Patterns of reticulation related to hybrid speciation and introgression do not fit traditional bifurcating models and are often more accurately represented by phylogenetic networks^87^. Therefore, we used a phylogenetic network approach that accounts for ILS through the coalescent model and for horizontal inheritance of genes through reticulation nodes in the network^78^. This maximum pseudolikelihood approach^88^ is implemented in PhyloNet v3.6.8^89^. A total of seven groups were defined in the MPL analyses: *R. melanophthalma*, *R. novomexicana*, *R. parilis, R. shushanii*, members of the ‘vagrant clade’, and members of the *R. porteri* group, along with the ‘saxicolous *haydenii*’ specimen, ‘715f’, with a clear mitonuclear conflict. In order to identify potential reticulations among more deeply diverged lineages, specimens recovered in the ‘vagrant clade’, and the *R. porteri* group were each treated as single groups. Based on the 407 CEG gene trees, the “InferNetwork_MPL” algorithm was run allowing for 1 through 5 reticulations by performing 1000 independent searches to avoid sampling in local optimums. Uncertain nodes were bypassed in the gene tree by applying a bootstrap support threshold of 50 using the -b flag. The 10 returned species networks were further optimized for both branch lengths and inheritance probabilities using a full likelihood framework by applying the -o flag. The best-fitting network was selected using the Akaike information criterion (AIC)^90,91^ by applying the number of parameters (*k*) as the number of branch lengths plus the number of reticulations with *L* as the likelihood value.

### Tests of interspecific gene flow

We used the program HyDe^92^ to detect potential interspecific hybridization using the SNP dataset constructed from the BUSCO gene regions. HyDe considers a root, four-taxon network consisting of an outgroup and a triplet of ingroup populations, P1, P2, and P3^93^ to detect hybridization from phylogenetic invariants that arise under the coalescent model with hybridization. Introgression between P3 and either P1 or P2 influences the relative frequencies of ABBA and BABA, and the D-statistic measures the imbalance between these frequencies^93^. We tested all possible triplet comparisons among species, treating *R. arbuscula*, *R. haydenii*, and the single specimen representing the ‘saxicolous *haydenii* population’ as separate species using the python script run_hyde.py. In all cases, *R. novomexicana* was treated as the outgroup. We only considered hypothesis tests that were significant at an overall α < 0.05 level (after incorporating a Bonferonni correction) with estimates of γ between 0 and 1. Z-scores greater than 3 are generally interpreted as strong evidence of introgression^94^.

### Using individual gene trees to infer mitochondrial introgression

To test if other samples from the ‘saxicolous *haydenii* population’ in western Montana (Supplementary Fig. S1) also exhibited patterns of mito-nuclear discordance, e.g., nuclear genome in the ‘vagrant clade’ and mitochondrial genome in the *R. porteri* group, we generated topologies from three short gene regions. Sequences from the three single-marker datasets—the nrDNA, the protein-coding region of HEC/Ndc80p, and the fragment of the mitochondrial genome—were aligned in MAFFT v7^74^. We implemented the G-INS-i alignment algorithm and ‘1PAM/K = 2’ scoring matrix, with an offset value of 0.2, and the remaining parameters were set to default values. The HEC/Ndc80p and mitochondrial datasets were represented by all metagenomic samples and sequences from the eight specimens of putative hybrid origin from the ‘saxicolous *haydenii* population’ generated using Sanger sequencing. ITS sequences were combined with 443 ITS sequences from Leavitt *et al*.^60^ to put our data within a broader specimen sampling context. Maximum likelihood topologies were reconstructed for each region using the program RAxML v8.2.10^95^ in the CIPRES Science Gateway server (http://www.phylo.org/portal2/). Substitution models for each locus were estimated using jModelTest v.2.1.10^96^, and nodal support was evaluated using 1000 bootstrap pseudo-replicates.

## Results

### Data and phylogenomic datasets

The most comprehensive nuclear dataset assembled using REALPHY included a total of 18,457,947 aligned nucleotide position characters. Metagenomic reads mapped to BUSCO genes larger than 1 kb resulted in an alignment of 1,620,052 bp; and from this alignment a total of 142,437 SNPs (polymorphic sites) were extracted. Our final CEG dataset comprised gene trees from 407 of the original 458 CEGs. The mitochondrial dataset generated using REALPHY included a total of 63,877 aligned nucleotide position characters. ITS, the HEC/Ndc80p protein, and the mitochondrial genome marker for the eight additional specimens of putative hybrid origin from western Montana, USA (Supplementary Fig. S2) were deposited in GenBank under accession numbers MN795100-MN795107. All alignments were deposited in FigShare (10.6084/m9.figshare).

### Phylogenomic reconstructions

**Figure 2:**
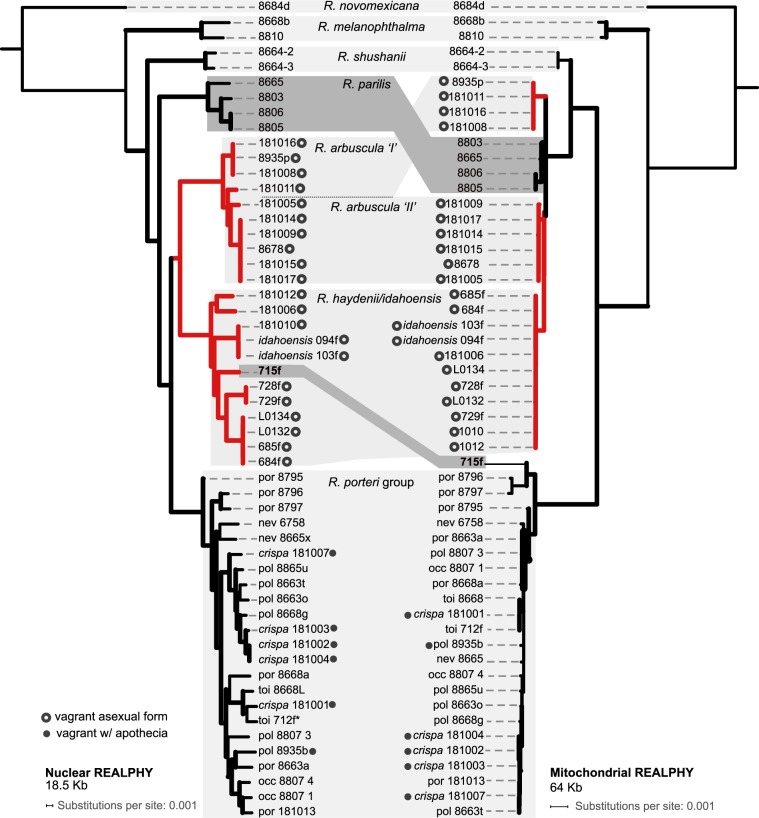
Topologies inferred from nuclear and mitochondrial REALPHY datasets. Thicker lines indicate 95+ bootstrap support. Corresponding clades in the nuclear and mitochondrial phylogenies are highlighted. Red branches indicate members of the nuclear ‘vagrant clade’, *R. arbuscula*, *R. haydenii*, and *R. idahoensis*, and the ‘saxicolous haydenii 715f’ specimen.

A well-supported phylogeny was inferred from the nuclear REALPHY dataset (Fig. 2). Vagrant forms representing *R. arbuscula*, *R. haydenii*, and *R. idahoensis* were recovered in a well-supported clade, the ‘vagrant clade’. Within the ‘vagrant clade’, two divergent, well-supported clades were recovered: one clade comprising specimens representing *R. arbuscula*, and the other comprised of specimens representing *R. haydenii*, *R. idahoensis*, and the ‘saxicolous *haydenii* 715f’ specimen from western Montana. Distinct clades within the ‘vagrant clade’ generally coincided with specimens collected from geographically distinct populations. Vagrant forms representing *R. melanophthalma* subsp. *cerebriformis* and *R. melanophthalma* subsp. *crispa* were recovered nested within the *R. porteri* group. Relationships among other *Rhizoplaca* species were congruent with previous studies based on genome-scale data^55,58^. Of the targeted vagrant taxa, *R. arbuscula*, *R. haydenii*, and *R. idahoensis*, only *R. arbuscula* was recovered as monophyletic (Fig. 2). We note that as in previous studies^55,58^, species within the *R. porteri* group, e.g., *R. occulta*, *R. polymorpha*, and *R. porteri*, were not recovered as monophyletic.

The phylogeny inferred from the mitochondrial REALPHY dataset recovered four distinct, major mitochondrial clades, with well-supported differences in phylogenetic relationships in comparison to relationships inferred from the nuclear phylogenomic dataset (Fig. 2). These four distinct clades were also recovered in exploratory phylogenetic analyses of the (i) concatenated CDS regions, (ii) concatenated intronic regions, and (iii) alignments of the complete mitochondrial contig, although relationships among major clades differed in each reconstruction (Supplementary Fig. S3). Of the four major mitochondrial clades, one was comprised of specimens representing *R. melanophthalma* (recovered in two separated clades in the CDS-derived phylogeny; Supplementary Fig. S3a); a second comprised *R. shushanii* specimens; the third was comprised of members of the *R. porteri* group, including *R. melanophthalma* subsp. *cerebriformis* and *R. melanophthalma* subsp. *crispa* and the ‘saxicolous haydenii 715f’ specimen from western Montana. The fourth clade included all vagrant forms representing *R. arbuscula*, *R. haydenii*, *R. idahoensis*, and the saxicolous species *R. parilis* (Fig. 2). Within this final clade, four distinct sub-clades were recovered, each corresponding to morphologically distinct specimens: (i) *R. haydenii* and *R. idahoensis* (vagrant forms), (ii) *R. arbuscula* specimens with narrow, finely dissected lobes (see Fig. 1a), (iii) *R. arbuscula* specimens with broader, more robust lobes more similar to *R. haydenii*, and (iv) *R. parilis* (an attached saxicolous species).

Discordance was observed between topologies inferred from the nuclear REALPHY and mitochondrial REALPHY datasets (Fig. 2). In the nuclear phylogeny, members of the ‘vagrant clade’ (*R. arbuscula*, *R. haydenii*, and *R. idahoensis*) were distinct from specimens representing *R. parilis*, in contrast to the mitochondrial phylogeny where members of the ‘vagrant clade’ were recovered within a single clade also comprised highly similar mitochondrial genomes from *R. parilis* specimens. Furthermore, the ‘saxicolous haydenii 715f’ specimen from western Montana, recovered in the ‘vagrant clade’ in the nuclear topology, showed evidence of mitochondrial introgression. Specifically, the ‘saxicolous haydenii 715f’ specimen was recovered among specimens representing the *R. porteri* group in the mitochondrial topology and not with other members of the ‘vagrant clade’ (Fig. 2).

**Figure 3:**
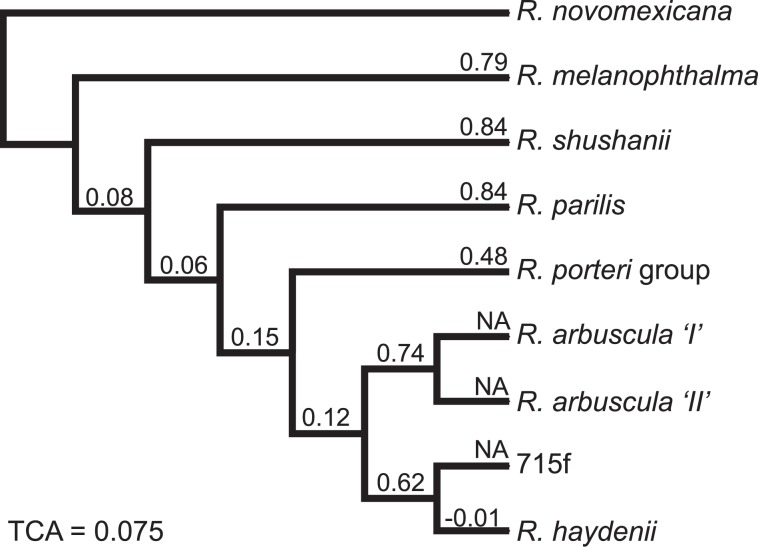
Species tree of the *R. melanophthalma* species complex inferred using ASTRAL-III and SVDquartet + PAUP*. Both species tree approaches inferred identical branching patterns and all nodes were recovered with 100% support. Values above branches correspond to internode certainty (IC) estimated from 407 individual BUSCO gene trees, reflecting the specific degree of incongruence for that internode (scaled between 0 and 1, values closer to 1 indicate no or limited conflict for a given internode, whereas values closer to 0 indicate increasing conflict). The relative tree certainty (TCA) is shown in the bottom left, characterizing the global degree of incongruence among individual gene trees.

Coalescent-based species tree analyses ASTRAL-III and SVDquartet + PAUP* generated identical branching patterns with bootstrap support values of 100% (Fig. 3). Relationships in species tree inferences were consistent with branching patterns inferred from the nuclear REALPHY datasets (Fig. 2). The TCA value (TCA = 0.075) revealed a pattern of genome-scale incongruence among trees, and the degree of incongruence for each internode in a set of gene trees, as determined using internode certainty values, is reported in Fig. 3. IC values indicated that most species level lineages were recovered as monophyletic in the majority of gene regions (Fig. 3), while relationships among these lineages had much lower IC values.

### Evidence of reticulated evolution

**Figure 4:**
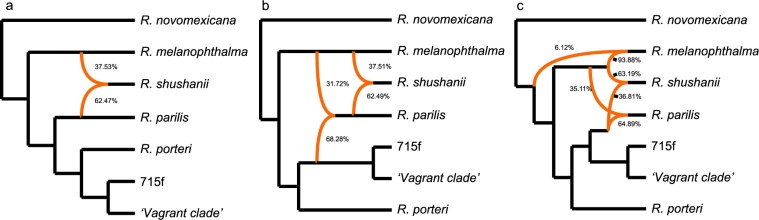
Phylogenetic networks inferred by PhyloNet using Maximum Pseudolikelihood under a one- (**a**), two-, (**b**), and three-reticulation model (**c**). AIC supported the three-reticulation model as the best-fitting scenario. Orange branches indicate lineages involved in reticulated histories; and numerical values are the inheritance probabilities for each reticulation.

MPL networks incorporating both incomplete lineage sorting and potential reticulations provided support for reticulate evolution in the *Rhizoplaca melanophthalma* species complex (Fig. 4). AIC supported the three-reticulation model as the best-fitting scenario (Table 2).

**Table 2:** PhyloNet results and AIC calculations to select optimal network, with the optimal network in bold.

# Reticulations	ln*L*	Δln*L*	# Branch lengths	*k*	AIC	ΔAIC
0	−3997622		7	7	7995257.07	8583.05408
1	−3995167	2454.80068	7	8	7990349.47	3675.45272
2	−3994063	1104.14995	7	9	7988143.17	1469.15281
**3**	−**3993325**	**737.576407**	**9**	**12**	**7986674.02**	**0**
4	−3993355	−29.658988	7	11	7986731.33	57.3179761
5	−3993584	−229.53622	9	14	7987196.41	522.390411

*L* is the likelihood value, and *k* is number of reticulations plus branch lengths (number of parameters used in the AIC calculation).

The one-, two- and three-reticulation MPL networks consistently inferred that *R. shushanii* has a hybrid origin resulting from reticulations between the *R. melanophthalma* and *R. parilis* lineages. The two-reticulation MPL network supports reticulations involving the ‘vagrant’ lineage with the *R. parilis* lineage, consistent with the inferred mitonuclear discordance (Fig. 2).

Sanger sequencing of the additional specimens of putative hybrid origin from western Montana (collected from the same population as specimen ‘saxicolous haydenii 715f’ [BRY-C]) also showed evidence of mitonuclear discordance (Supplementary Fig. S2a–c). Relationships of five of the eight additional samples from the putative hybrid population were consistent with the single specimen included in the phylogenomic portion of the study, while the remaining three specimens showed slightly different patterns of mitonuclear discordance. In the nuclear ITS topology, specimens were recovered in two separate clades—the *R. melanophthalma* clade which occurred as sister to the second clade comprised of *R. arbuscula, R. haydenii*, *R. idahoensis*, and *R. parilis*. In the topology inferred from the single-copy nuclear HEC/Ndc80p loci, specimens were recovered in two distinct clades: one group within the *‘haydenii*/*idahoensis*’ clade and the other clade comprised exclusively of the three putative hybrid species with an unresolved relationship to other clades. In the topology inferred from mitochondrial marker, the putative hybrid specimens from western Montana were all recovered in the mitochondrial *R. porteri* group but in three distinct groups (Supplementary Fig. S2a–c).

The HyDe analysis revealed 24 significant triplet comparisons supporting hybridization, including seven comparisons with Z-scores >3 (Table 3). *Rhizoplaca shushanii* was consistently inferred to be of hybrid origin with the highest Z-scores. *Rhizoplaca* species from the ‘vagrant clade’ were also consistently inferred to have been involved in hybridization, particularly individuals representing *R. arbuscula* (Table 3).

**Table 3:** HyDe inferences of hybridization in the *Rhizoplaca melanophthalma* species complex.

P1	Hybrid	P2	Z-score	P-value	Gamma
*R. arbuscula*	*R. shushanii*	*R. melanophthalma*	**13.49**	0	0.81
*R. parilis*	*R. shushanii*	*R. melanophthalma*	**13.15**	0	0.81
*R. melanophthalma*	*R. shushanii*	*R. porteri*	**12.86**	0	0.82
*R. haydenii*	*R. shushanii*	*R. melanophthalma*	**11.74**	0	0.82
*R*. ‘715f’	*R. shushanii*	*R. melanophthalma*	**11.26**	0	0.19
*R. arbuscula*	*R. haydenii*	*R. parilis*	**5.14**	1.35E-07	0.90
*R. arbuscula*	*R. haydenii*	*R. porteri*	**5.01**	2.67E-07	0.77
*R. arbuscula*	*R. haydenii*	*R*. ‘715f’	2.82	0.002	0.03
*R. shushanii*	*R. arbuscula*	*R*. ‘715f’	2.81	0.002	0.04
*R. arbuscula*	*R*. ‘715f’	*R. parilis*	2.79	0.003	0.93
*R. arbuscula*	*R*. ‘715f’	*R. porteri*	2.48	0.007	0.85
*R. shushanii*	*R. arbuscula*	*R. haydenii*	2.45	0.007	0.03
*R. arbuscula*	*R*. ‘715f’	*R. melanophthalma*	2.39	0.008	0.98
*R. arbuscula*	*R. parilis*	*R. parilis*	2.38	0.009	0.91
*R. parilis*	*R. haydenii*	*R. porteri*	2.27	0.012	0.08
*R. arbuscula*	*R. haydenii*	*R. melanophthalma*	2.23	0.013	0.98
*R. shushanii*	*R. parilis*	*R. haydenii*	2.22	0.013	0.10
*R. parilis*	*R*. ‘715f’	*R. melanophthalma*	2.22	0.013	0.97
*R. shushanii*	*R. arbuscula*	*R. porteri*	2.18	0.015	0.05
*R. shushanii*	*R. parilis*	*R*. ‘715f’	2.08	0.019	0.10
*R. shushanii*	*R. parilis*	*R. porteri*	2	0.023	0.10
*R. melanophthalma*	*R*. ‘715f’	*R. porteri*	1.9	0.029	0.02
*R*. ‘715f’	*R. haydenii*	*R. porteri*	1.79	0.037	0.98
*R. parilis*	*R. haydenii*	*R*. ‘715f’	1.72	0.043	0.01

Only putative hybridization events with P-values <0.05 are indicated; results are sorted according the highest Z-scores, and Z-scores >3 are in bold text. ‘P1’ corresponds to an initial donor species, ‘Hybrid’ represents the species containing putatively introgressed loci, and ‘P2’ is a second hybridizing donor. The gamma parameter is an estimate the amount of admixture/introgression from P2 into the hybrid.

## Discussion

Recently, the roles of hybridization have been highlighted as important mechanisms for facilitating rapid speciation and adaptive radiations^97^. In particular, hybridization between early branching lineages can augment standing variation that contributes to later speciation events^4,98–102^. Similarly, our results support the potential role of genomic combinatorial mechanisms in the diversification of symbiotic fungi. Mitonuclear discordance in members of the *R. melanophthalma* complex (Fig. 2), ABBA-BABA tests for introgression (Table 3), and reticulated evolutionary histories inferred from nuclear phylogenomic data (Fig. 4) provide evidence that hybridization played a central role in the diversification of this group. While robust phylogenies were inferred from the nuclear phylogenomic datasets (Figs. 2 and 3), the evolutionary history of the mitochondrial genome appears to be more complex in this group. As has been demonstrated in other studies, mitochondrial genome evolution in fungi is complex^103,104^. Mitochondrial genome evolution in the *R. melanophthalma* species complex also appears to be affected by dynamic evolutionary processes, with different phylogenetic relationships inferred from different portions of the mitochondrial genome (Supplementary Fig. 3). Additional research will be required to appropriately characterize features of mitochondrial genome evolution, e.g., intron gains/losses, gene rearrangement, recombination, etc., in lichen-forming fungi.

These data support a complex model of speciation in the *Rhizoplaca melanophthalma* group with multiple reticulation events throughout its diversification history. Despite the high internode certainty values and strongly supported phylogenetic clades corresponding to species in the *R. melanophthalma* species complex (considering members of the *R. porteri* group as a single species), our results indicated significant evidence for allele sharing in the diversification history of the group. These results are congruent with an interpretation that hybridization may introduce reproductive barriers, and hybrid lineages become reproductively isolated with limited or no ongoing gene flow once established, e.g., consistent genome-wide support for species lineages but significant genome-scale conflict for relationships among lineages.

Pure hybrids are rarely encountered due to the genetic incompatibilities that reduce fitness^3^, but sometimes parental genomes are compatible enough for the persistence of a new hybrid species, either in a new niche or with high enough fitness to compete with the parent species^97^. In the *R. melanophthalma* species complex, our PhyloNet analysis indicated that *R. shushanii* is potentially the result of a hybrid speciation event between the *R. melanophthalma* and *R. parilis* lineages, or at least introgressed with ghost lineages, leading to what is now considered a distinct species. These results are further supported by inferred hybrid origin of *R. shushanii* with high associated Z-scores in the HyDe analysis (Table 3). However, we note that PhyloNet and HyDe tests provided somewhat contrasting inferences for the parental lineages involved in the reticulated evolutionary history in this complex (Fig. 4 and Table 3). While additional work will be required to more fully elucidate the timing, direction, and lineages involved in the reticulated evolutionary history of the *R. melanophthalma* group, our study emphasizes the importance of considering hybridization in an organism’s evolutionary history, even when relationships are strongly supported as bifurcating in traditional phylogenomic analyses. Furthermore, by using multiple analytical tools for inferring introgression, operating under different assumptions, e.g., MPL & ABBA/BABA test of introgression, and then viewing congruence between methods can provide greater support for the role of reticulation in the evolutionary history of the targeted group^105^.

To date, *R. shushanii* (Fig. 1e) has only been found at subalpine sites in southern Utah despite worldwide sampling efforts, while *R. melanophthalma* and *R. parilis* can be found worldwide^50^. Perhaps the hybrid speciation of *R. shushanii* occurred only in places where there was a suitable niche available. Schumer *et al*.^8^ suggests that in order to have evidence of true hybrid speciation, it is necessary to show that hybridization leads to reproductive isolation. This criterion is not readily applicable to fungi, as they can speciate without complete reproductive isolation^36,106^. Furthermore, hybridization and reproductive isolation are especially difficult to account for with lichen-forming fungi, which cannot be easily cultured *in vitro* for experimental testing. Regardless, there is value in accounting for gene flow and utilizing phylogenetic networks to explore evolutionary relationships.

A number of studies show that hybrid sterility develops as a result of incompatibilities in the genome^21,107,108^. As many species of lichen-forming fungi can reproduce both sexually and asexually, it’s possible that the evolution of hybrid sterility could trigger a lichen-forming fungus to rely primarily on asexual reproduction. While *R. parilis* is abundantly fertile, members of the ‘vagrant clade’ with the putative introgressed *R. parilis* mitochondrial genomes are strictly asexual. We speculate that a mitonuclear incompatibility may have contributed to the differences in reproductive strategies. We note that one single member of the nuclear ‘vagrant clade’, the ‘saxicolous haydenii 715f’ specimen, along with the five of the eight specimens also collected from the same population in western Montana (Supplementary Fig. S2), appear to have an introgressed mitochondrial genome more similar to those from the *R. porteri* group. These six specimens differ morphologically from other members of the *R. ‘haydenii*’ clade, in that like other members of the *R. melanophthalma* complex, they are umbilicate and attached to rocks. None of the specimens recovered within the ‘vagrant clade’ using the nuclear phylogenomic dataset and with ‘*porteri*’-type mitochondrial genomes produce normal, fully developed apothecia, although it appears that stunted or partially developed apothecia occurred on these thalli (Fig. 1c). However, investigating mating-type loci on the population level could provide additional insights. Pizarro *et al*.^109^ recently found a representative from the *Rhizoplaca melanopthalma* complex to be heterothallic, with a loss of primary homothallism in the class Lecanoromycetes. An imbalance of mating types on the population level could paint the picture of why, evolutionarily, these lichen-forming fungi might have shifted to asexuality.

Our study also sheds light on how asexual organisms might fit into the evolutionary picture—how they might come to be and how genetically isolated they truly are. Our results reveal significant phylogeographic structure in the asexual *R. haydenii/idahoensis* clade (Fig. 2), suggesting that populations in this lineage may not rely exclusively on clonal reproduction. Other studies have revealed evidence of recombination in what were thought to be asexual lichens^110,111^. It has been argued that fungal sexuality should be viewed as a spectrum, where obligate sexual reproduction and obligate asexual reproduction in a single species rarely occur^44,112,113^. It is also possible for fungi to exchange genetic material via fusion of vegetative cells^3,114,115^. Alternatively, this phylogeographic structure may have arisen from variation acquired before the loss of sexuality, or these distinct lineages may arise from independent hybridization events. It is also possible this lineage is a result of an ancient hybridization event, and local populations have since fixed their own unique genetic properties.

Hybridization can generate novel phenotypes, which can sometimes manifest later in evolutionary history under new selective pressures^97^. We originally hypothesized that hybridization had played a role in the evolution of vagrancy within the *R. melanophthalma* species complex, as demonstrated by the distinctly vagrant, asexual *R. haydenii* clade. We found that hybridization doesn’t fully explain vagrant forms. Vagrants (erratic vs. obligate forms) in the *R. porteri* group, e.g., *R. melanophthalma* subsp. *crispa*, did not show evidence of mitochondrial introgression, in contrast to the vagrant taxa (*R. arbuscula, R. haydenii*, and *R. idahoensis*) with *R. parilis*. However, those with the strongest evidence for hybridization showed the most morphological distinctiveness, e.g., *R. shushanii* and members of the ‘vagrant clade’. In future research, it would be worthwhile to investigate the influence of the nonfungal symbionts on the evolution of vagrancy, as symbionts can also influence lichen morphology^116,117^.

In conclusion, by analyzing phylogenomic data from both mitochondrial and nuclear genomes of species within the *Rhizoplaca melanophthalma* species complex and by reconstructing a phylogenetic network, we showed evidence of a mitonuclear discordance, as well as a reticulated evolutionary history within the complex. Our results support the need for considering reticulate phylogenies when investigating species boundaries and evolutionary history, even in well-supported topologies inferred from genome-scale data. Similarly, while the role of hybridization and recombination has long been considered in diversification of lichen-forming fungi^33,41–43^, our results provide important impetus for explicitly considering hybridization/introgression in process of speciation in symbiotic fungi and their potential role in generating novel phenotypes and introducing reproductive barriers.

## Supplementary information

Supporting Information.

Supporting Information 2.

Supporting Information 3.

Supporting Information 4.

## Data Availability

Raw sequence data generated for this study were deposited in NCBI’s Short Read Archive under project PRJNA576709. RealPhy alignments, both nuclear and mitochondrial DNA will be available on FigShare (10.6084/m9.figshare.11299040). Sanger sequences are deposited in GenBank, under accession Nos. MN795100-MN795107.

## References

[1] Chan KMA, Levin SA (2005). Leaky prezygotic isolation and porous genomes: Rapid introgression of maternally inherited DNA. Evolution.

[2] Mallet J (2005). Hybridization as an invasion of the genome. Trends Ecol. Evol..

[3] Stukenbrock EH (2016). The Role of Hybridization in the Evolution and Emergence of New Fungal Plant Pathogens. Phytopathology.

[4] Hedrick PW (2013). Adaptive introgression in animals: examples and comparison to new mutation and standing variation as sources of adaptive variation. Mol. Ecol..

[5] Hill GE (2017). The mitonuclear compatibility species concept. Auk.

[6] Huang JP (2016). Parapatric genetic introgression and phenotypic assimilation: testing conditions for introgression between Hercules beetles (*Dynastes*, Dynastinae). Mol. Ecol..

[7] Feliner GN (2017). Is homoploid hybrid speciation that rare? An empiricist’s view. Heredity.

[8] Schumer M, Rosenthal GG, Andolfatto P (2014). How common is homoploid hybrid speciation?. Evolution.

[9] Abbott R (2013). Hybridization and speciation. J. Evolut. Biol..

[10] Chapman MA, Burke JM (2007). Genetic divergence and hybrid speciation. Evolution.

[11] Tigano A, Friesen VL (2016). Genomics of local adaptation with gene flow. Mol. Ecol..

[12] Rieseberg LH (2003). Major ecological transitions in wild sunflowers facilitated by hybridization. Sci..

[13] Gladieux P (2014). Fungal evolutionary genomics provides insight into the mechanisms of adaptive divergence in eukaryotes. Mol. Ecol..

[14] Dasmahapatra KK (2012). Butterfly genome reveals promiscuous exchange of mimicry adaptations among species. Nat..

[15] Lamichhaney S (2018). Rapid hybrid speciation in Darwin’s finches. Sci..

[16] Stankowski Sean, Streisfeld Matthew A. (2015). Introgressive hybridization facilitates adaptive divergence in a recent radiation of monkeyflowers. Proceedings of the Royal Society B: Biological Sciences.

[17] Fishman, L. & Sweigart, A. L. When Two Rights Make a Wrong: The Evolutionary Genetics of Plant Hybrid Incompatibilities, Vol. 69 *Annual Review of Plant Biology* (ed S. S. Merchant) 707–731 (2018). 10.1146/annurev-arplant-042817-040113 29505737

[18] Mack KL, Nachman MW (2017). Gene regulation and speciation. Trends Genet..

[19] Bonnet T, Leblois R, Rousset F, Crochet PA (2017). A reassessment of explanations for discordant introgressions of mitochondrial and nuclear genomes. Evolution.

[20] Burton RS, Barreto FS (2012). A disproportionate role for mtDNA in Dobzhansky-Muller incompatibilities?. Mol. Ecol..

[21] Lee HY (2008). Incompatibility of nuclear and mitochondrial genomes causes hybrid sterility between two yeast species. Cell.

[22] Sloan DB, Havird JC, Sharbrough J (2017). The on-again, off-again relationship between mitochondrial genomes and species boundaries. Mol. Ecol..

[23] Toews DP, Brelsford A (2012). The biogeography of mitochondrial and nuclear discordance in animals. Mol. Ecol..

[24] Ivanov V, Lee KM, Mutanen M (2018). Mitonuclear discordance in wolf spiders: Genomic evidence for species integrity and introgression. Mol. Ecol..

[25] Giordano L, Sillo F, Garbelotto M, Gonthier P (2018). Mitonuclear interactions may contribute to fitness of fungal hybrids. Sci. Rep..

[26] Greig D, Louis EJ, Borts RH, Travisano M (2002). Hybrid speciation in experimental populations of yeast. Sci..

[27] Anderson JB (2003). Mode of selection and experimental evolution of antifungal drug resistance in *Saccharomyces cerevisiae*. Genet..

[28] Stukenbrock EH, Christiansen FB, Hansen TT, Dutheil JY, Schierup MH (2012). Fusion of two divergent fungal individuals led to the recent emergence of a unique widespread pathogen species. Proc. Natl Acad. Sci. USA.

[29] Greenspan SE (2018). Hybrids of amphibian chytrid show high virulence in native hosts. Sci. Rep..

[30] Silva DN, Varzea V, Paulo OS, Batista D (2018). Population genomic footprints of host adaptation, introgression and recombination in coffee leaf rust. Mol. Plant. Pathol..

[31] Anderson E, Rudolph ED (1956). An analysis of variation in a variable population of *Cladonia*. Evolution.

[32] Culberson CF, Culberson WL, Johnson A (1988). Gene Flow in Lichens. Am. J. Botany.

[33] O’Brien H, Miadlikowska J, Lutzoni F (2009). Assessing Reproductive Isolation in Highly Diverse Communities of the Lichen-Forming Fungal Genus *Peltigera*. Evolution.

[34] Zoller S, Lutzoni F, Scheidegger C (1999). Genet. Var. Popul. threatened lichen Lobaria pulmonaria Switz. Implic. its conservation..

[35] Magain N, Sérusiaux E, Zhurbenko MP, Lutzoni F, Miadlikowska J (2016). Disentangling the *Peltigera polydactylon* Species Complex by Recognizing Two New Taxa, *P. polydactylon* subsp. *udeghe* and *P. seneca*. Herzogia.

[36] Steenkamp ET, Wingfield MJ, McTaggart AR, Wingfield BD (2018). Fungal species and their boundaries matter - Definitions, mechanisms and practical implications. Fungal Biol. Rev..

[37] Prieto María, Wedin Mats (2013). Dating the Diversification of the Major Lineages of Ascomycota (Fungi). PLoS ONE.

[38] Lucking R, Huhndorf S, Pfister DH, Plata ER, Lumbsch HT (2009). Fungi evolved right on track. Mycologia.

[39] Lumbsch HT, Leavitt SD (2011). Goodbye morphology? A paradigm shift in the delimitation of species in lichenized fungi. Fungal Diversity.

[40] Culberson CF, Hale ME (1973). Chemical and morphological evolution in *Parmelia* sect. *Hypotrachyna*: Product of ancient hybridization?. Brittonia.

[41] Ertz D (2009). Towards a new classification of the *Arthoniales* (*Ascomycota*) based on a three-gene phylogeny focussing on the genus *Opegrapha*. Mycol. Res..

[42] Ekman S, Fröberg L (1988). Taxonomical problems in *Aspicilia contorta* and *A. hoffmannii* - an effect of hybridization?. Int. J. Mycology Lichenology.

[43] Widhelm TJ (2019). Multiple historical processes obscure phylogenetic relationships in a taxonomically difficult group (Lobariaceae, Ascomycota). Sci. Rep..

[44] Tripp EA, Lendemer JC (2017). Twenty-seven modes of reproduction in the obligate lichen symbiosis. Brittonia.

[45] Murtagh GJ, Dyer PS, Crittenden PD (2000). Reproductive systems: Sex and the single lichen. Nat..

[46] Billiard S, Lopez-Villavicencio M, Hood ME, Giraud T (2012). Sex, outcrossing and mating types: unsolved questions in fungi and beyond. J. Evol. Biol..

[47] Taylor JW, Jacobson DJ, Fisher MC (1999). The Evolution of Asexual Fungi: Reproduction, Speciation and Classification. Annu. Rev. Phytopathol..

[48] Wilson AM (2015). Homothallism: an umbrella term for describing diverse sexual behaviours. IMA Fungus.

[49] Schardl CL, Craven KD (2003). Interspecific hybridization in plant-associated fungi and oomycetes: a review. Mol. Ecol..

[50] Leavitt S (2013). DNA barcode identification of lichen-forming fungal species in the *Rhizoplaca melanophthalma* species-complex (Lecanorales, Lecanoraceae), including five new species. MycoKeys.

[51] Leavitt SD (2011). Complex patterns of speciation in cosmopolitan “rock posy” lichens–discovering and delimiting cryptic fungal species in the lichen-forming *Rhizoplaca melanophthalma* species-complex (Lecanoraceae, Ascomycota). Mol. Phylogenet Evol..

[52] Leavitt SD (2013). Local representation of global diversity in a cosmopolitan lichen-forming fungal species complex (*Rhizoplaca*, Ascomycota). J. Biogeography.

[53] Rosentreter R (1993). Vagrant Lichens in North America. Bryologist.

[54] Feurtey A, Stukenbrock EH (2018). Interspecific Gene Exchange as a Driver of Adaptive Evolution in Fungi. Annu. Rev. Microbiol..

[55] Leavitt SD (2016). Resolving evolutionary relationships in lichen-forming fungi using diverse phylogenomic datasets and analytical approaches. Sci. Rep..

[56] Leavitt SD (2013). DNA barcode Identif. lichen-forming fungal species Rhizoplaca melanophthalma species-complex, including five N. species MycoKeys.

[57] Leavitt Steven D., Keuler Rachel, Newberry Clayton C., Rosentreter Roger, Clair Larry L. St. (2019). Shotgun sequencing decades-old lichen specimens to resolve phylogenomic placement of type material. Plant and Fungal Systematics.

[58] Grewe F, Huang JP, Leavitt SD, Lumbsch HT (2017). Reference-based RADseq resolves robust relationships among closely related species of lichen-forming fungi using metagenomic DNA. Sci. Rep..

[59] McCune, B. & Rosentreter, R. *Biotic Soil Crust Lichens of the Columbia Basin*. Vol. 39 (Northwest Lichenologists, 2007).

[60] Leavitt SD (2016). Cryptic diversity and symbiont interactions in rock-posy lichens. Mol. Phylogenet Evol..

[61] Schoch CL (2012). Nuclear ribosomal internal transcribed spacer (ITS) region as a universal DNA barcode marker for Fungi. Proc. Natl Acad. Sci. USA.

[62] Simao FA, Waterhouse RM, Ioannidis P, Kriventseva EV, Zdobnov EM (2015). BUSCO: assessing genome assembly and annotation completeness with single-copy orthologs. Bioinforma..

[63] Bertels F, Silander OK, Pachkov M, Rainey PB, van Nimwegen E (2014). Automated reconstruction of whole-genome phylogenies from short-sequence reads. Mol. Biol. Evol..

[64] Zeng Q (2018). Comparative genomics of Spiraeoideae-infecting *Erwinia amylovora* strains provides novel insight to genetic diversity and identifies the genetic basis of a low-virulence strain. Mol. Plant. Pathol..

[65] Langmead B, Salzberg SL (2012). Fast gapped-read alignment with Bowtie 2. Nat. Methods.

[66] Stanke M, Steinkamp R, Waack S, Morgenstern B (2004). AUGUSTUS: a web server for gene finding in eukaryotes. Nucleic Acids Res..

[67] Merchant Nirav, Lyons Eric, Goff Stephen, Vaughn Matthew, Ware Doreen, Micklos David, Antin Parker (2016). The iPlant Collaborative: Cyberinfrastructure for Enabling Data to Discovery for the Life Sciences. PLOS Biology.

[68] Goff SA (2011). The iPlant Collaborative: Cyberinfrastructure for Plant Biology. Front. Plant. Sci..

[69] Misof B (2013). Selecting informative subsets of sparse supermatrices increases the chance to find correct trees. BMC Bioinforma..

[70] Parra G, Bradnam K, Korf I (2007). CEGMA: a pipeline to accurately annotate core genes in eukaryotic genomes. Bioinforma..

[71] Edgar RC (2004). MUSCLE: multiple sequence alignment with high accuracy and high throughput. Nucleic Acids Res..

[72] Bankevich A (2012). SPAdes: a new genome assembly algorithm and its applications to single-cell sequencing. J. Comput. Biol..

[73] Wyman SK, Jansen RK, Boore JL (2004). Automatic annotation of organellar genomes with DOGMA. Bioinforma..

[74] Katoh K, Standley DM (2013). MAFFT multiple sequence alignment software version 7: improvements in performance and usability. Mol. Biol. Evol..

[75] Tonini, J., Moore, A., Stern, D., Shcheglovitova, M. & Orti, G. Concatenation and species tree methods exhibit statistically indistinguishable accuracy under a range of simulated conditions. *PLoS Curr* **7**, 10.1371/currents.tol.34260cc27551a527b124ec5f6334b6be (2015). 10.1371/currents.tol.34260cc27551a527b124ec5f6334b6be PMC4391732 25901289

[76] Zhang C, Rabiee M, Sayyari E, Mirarab S (2018). ASTRAL-III: polynomial time species tree reconstruction from partially resolved gene trees. BMC Bioinforma..

[77] Chifman J, Kubatko L (2014). Quartet inference from SNP data under the coalescent model. Bioinforma..

[78] Solis-Lemus C, Ane C (2016). Inferring phylogenetic networks with maximum pseudolikelihood under incomplete lineage sorting. PLoS Genet..

[79] Nguyen LT, Schmidt HA, von Haeseler A, Minh BQ (2015). IQ-TREE: a fast and effective stochastic algorithm for estimating maximum-likelihood phylogenies. Mol. Biol. Evol..

[80] Hoang DT, Chernomor O, von Haeseler A, Minh BQ, Vinh LS (2018). UFBoot2: Improving the Ultrafast Bootstrap Approximation. Mol. Biol. Evol..

[81] Kalyaanamoorthy S, Minh BQ, Wong TKF, von Haeseler A, Jermiin LS (2017). ModelFinder: fast model selection for accurate phylogenetic estimates. Nat. Methods.

[82] Edwards SV (2009). Is a new and general theory of molecular systematics emerging?. Evolution.

[83] Chou Jed, Gupta Ashu, Yaduvanshi Shashank, Davidson Ruth, Nute Mike, Mirarab Siavash, Warnow Tandy (2015). A comparative study of SVDquartets and other coalescent-based species tree estimation methods. BMC Genomics.

[84] Sayyari E, Mirarab S (2016). Fast coalescent-based computation of local branch support from quartet frequencies. Mol. Biol. Evolution.

[85] Swofford, D. *PAUP*. Phylogenetic Analysis Using Parsimony (*and Other Methods). Version 4.0b10*. Vol. Version 4.0 (2002).

[86] Salichos L, Stamatakis A, Rokas A (2014). Novel information theory-based measures for quantifying incongruence among phylogenetic trees. Mol. Biol. Evol..

[87] Burbrink FT, Gehara M (2018). The Biogeography of Deep Time Phylogenetic Reticulation. Syst. Biol..

[88] Yu, Y. & Nakhleh, L. A maximum pseudo-likelihood approach for phylogenetic networks. *Bmc Genomics* **16**, 10.1186/1471-2164-16-s10-s10 (2015). 10.1186/1471-2164-16-S10-S10 PMC4602316 26450642

[89] Wen DQ, Yu Y, Zhu JF, Nakhleh L (2018). Inferring phylogenetic networks using PhyloNet. Syst. Biol..

[90] Sullivan J, Joyce P (2005). Model Selection in Phylogenetics. Annu. Rev. Ecology, Evolution, Syst..

[91] Akaike, H. In *Selected Papers of* Hirotugu Akaike (eds Emanuel Parzen, Kunio Tanabe, & Genshiro Kitagawa) 199–213 (Springer New York, 1998).

[92] Blischak PD, Chifman J, Wolfe AD, Kubatko LS (2018). HyDe: A Python Package for Genome-Scale Hybridization Detection. Syst. Biol..

[93] Green RE (2010). A Draft Sequence of the Neandertal Genome. Sci..

[94] Eaton DAR, Ree RH (2013). Inferring Phylogeny and Introgression using RADseq Data: An Example from Flowering Plants (Pedicularis: Orobanchaceae). Syst. Biol..

[95] Stamatakis A (2014). RAxML version 8: a tool for phylogenetic analysis and post-analysis of large phylogenies. Bioinforma..

[96] Darriba D, Taboada GL, Doallo R, Posada D (2012). jModelTest 2: more models, new heuristics and parallel computing. Nat. Methods.

[97] Marques David A., Meier Joana I., Seehausen Ole (2019). A Combinatorial View on Speciation and Adaptive Radiation. Trends in Ecology & Evolution.

[98] Lewontin RC (1966). Hybridization as a new source of variation for adaptation to new environments. Evolution.

[99] Soltis DE (2004). Recent and recurrent polyploidy in *Tragopogon* (Asteraceae): cytogenetic, genomic and genetic comparisons. Biol. J. Linn. Soc..

[100] Wallbank RW (2016). Evolutionary novelty in a butterfly wing pattern through enhancer shuffling. PLoS Biol..

[101] Bell CD (2012). Rapid diversification of *Tragopogon* and ecological associates in Eurasia. J. Evol. Biol..

[102] Bassham S, Catchen J, Lescak E, von Hippel FA, Cresko WA (2018). Repeated Selection of Alternatively Adapted Haplotypes Creates Sweeping Genomic Remodeling in Stickleback. Genet..

[103] Pogoda CS (2019). Genome streamlining via complete loss of introns has occurred multiple times in lichenized fungal mitochondria. Ecol. Evol..

[104] Aguileta G (2014). High variability of mitochondrial gene order among fungi. Genome Biol. Evol..

[105] Blair, C. & Ane, C. Phylogenetic trees and networks can serve as powerful and complementary approaches for analysis of genomic data. *Syst Biol*, 10.1093/sysbio/syz056 (2019). 10.1093/sysbio/syz056 31432090

[106] Kohn LM (2005). Mechanisms of fungal speciation. Annu. Rev. Phytopathol..

[107] Lodé T (2012). Adaptive Significance and Long-Term Survival of Asexual Lineages. Evolut. Biol..

[108] Janko K (2018). Hybrid asexuality as a primary postzygotic barrier between nascent species: On the interconnection between asexuality, hybridization and speciation. Mol. Ecol..

[109] Pizarro D (2019). Whole-Genome Sequence Data Uncover Widespread Heterothallism in the Largest Group of Lichen-Forming Fungi. Genome Biol. Evol..

[110] Kroken S, Taylor JW (2001). Outcrossing and recombination in the lichenized fungus *Letharia*. Fungal Genet. Biol..

[111] Buschbom J, Mueller GM (2006). Testing “species pair” hypotheses: evolutionary processes in the lichen-forming species complex *Porpidia flavocoerulescens* and *Porpidia melinodes*. Mol. Biol. Evol..

[112] Honegger R, Zippler U (2007). Mating systems in representatives of Parmeliaceae, Ramalinaceae and Physciaceae (Lecanoromycetes, lichen-forming ascomycetes). Mycol. Res..

[113] Tripp EA (2016). Is asexual reproduction an evolutionary dead end in lichens?. Lichenologist.

[114] Roper M, Ellison C, Taylor JW, Glass NL (2011). Nuclear and genome dynamics in multinucleate ascomycete fungi. Curr. Biol..

[115] Clutterbuck AJ (1996). Parasexual recombination in fungi. Indian. Acad. Sci..

[116] Ertz D, Guzow-Krzeminska B, Thor G, Lubek A, Kukwa M (2018). Photobiont switching causes changes in the reproduction strategy and phenotypic dimorphism in the Arthoniomycetes. Sci. Rep..

[117] Spribille T (2018). Relative symbiont input and the lichen symbiotic outcome. Curr. Opin. Plant. Biol..

