# Supplementary material for: Genome-scale data reveal the role of hybridization in lichen-forming fungi
Source: Sci Rep. 2020 Jan 30;10:1497. doi: 10.1038/s41598-020-58279-x (PMC6992703; doi:10.1038/s41598-020-58279-x)

Genome-scale data reveal the role of hybridization in lichen-forming fungi

Supplementary Information

Rachel Keuler[1], Alexis Garretson[1], Theresa Saunders[1], Robert Erickson[1], Nathan St. Andre[1], Felix Grewe[2], Hayden Smith[1], H. Thorsten Lumbsch[2], Jen-Pan Huang[3], Larry L. St. Clair[1,4], Steven D. Leavitt[1,4,*]

[1]*Department of Biology, Brigham Young University, 4102 Life Science Building, Provo, UT 84602, U.S.A.*
[2]*Grainger Bioinformatics Center, Science & Education, The Field Museum, 1400 S. Lake Shore Drive, Chicago, IL 60605, U.S.A.*
[3]*Biodiversity Research Center, Academia Sinica, 128 Academia Rd, Section 2, Nankang District, Taipei 11529, Taiwan*
[4]*M. L. Bean Life Science Museum, Brigham Young University, 1115 MLBM, Provo, UT 84602, U.S.A.*
[*]Corresponding author's e-mail: steve_leavitt@byu.edu

Figure S2: Single locus phylogenies assessing mitonuclear discordance in members of the 'saxicolous *haydenii* population'. Topologies were inferred using the (a) the ITS region, (b) a fragment of the HEC/Ndc80p protein-coding gene region; and (c) a short region of the mitochondrial genome. Specimens from the 'saxicolous *haydenii* population' are labeled '715a' – '715i'.

Supplementary Fig. S2a. ITS topology.

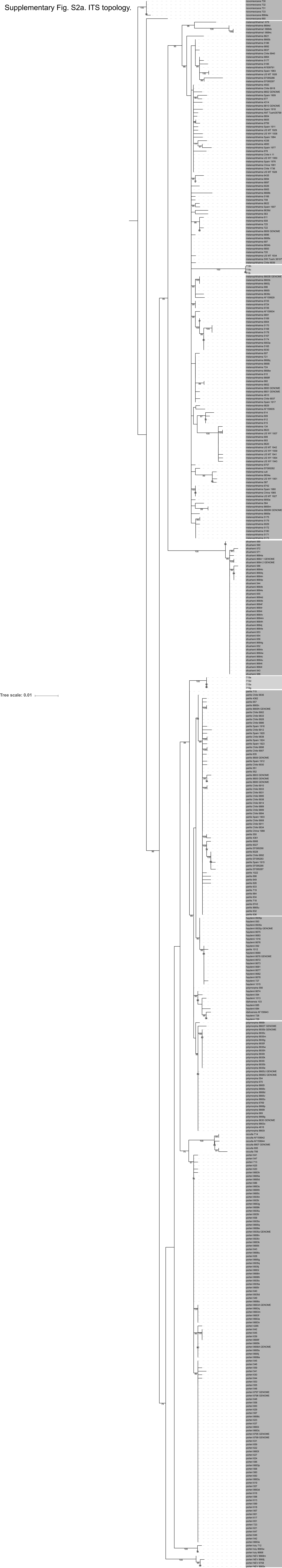

Supplementary Fig. S2b. HEC/Ndc80p topology

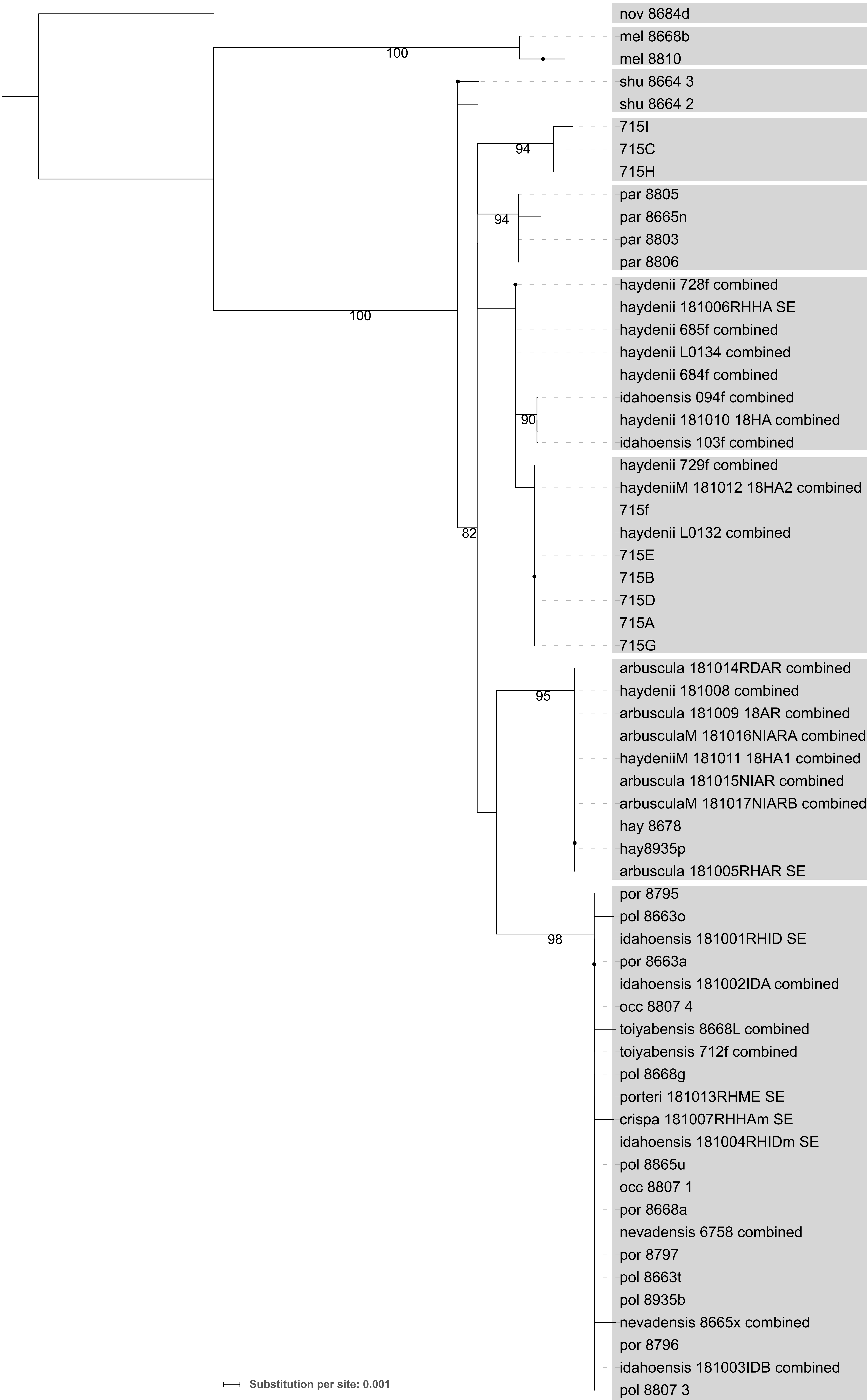

Supplementary Fig. S2c. mitochondrial marker topology

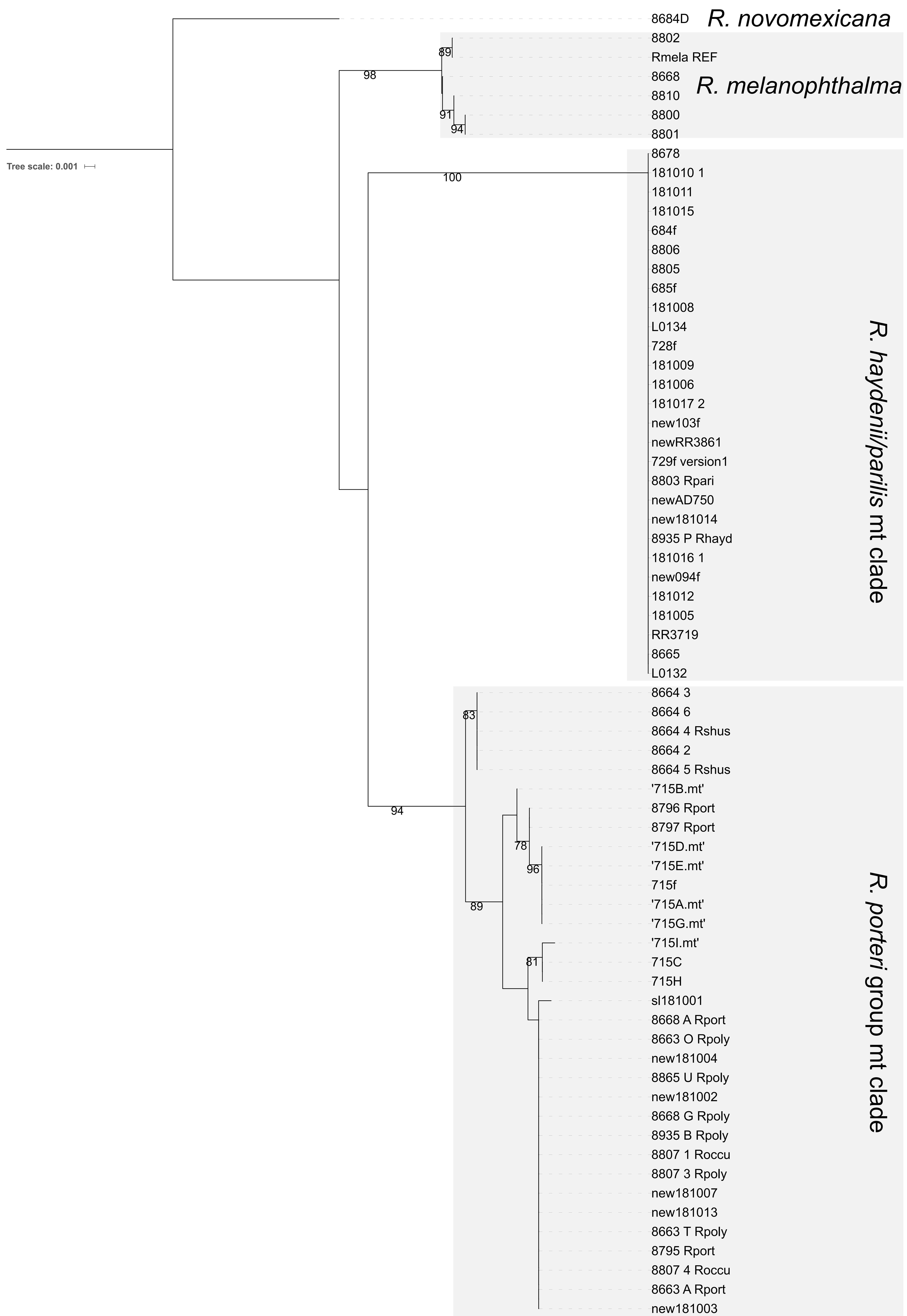

Supplement: Supplementary file 3 — Supporting Information 3. [file 41598_2020_58279_MOESM3_ESM.pdf]
