# Supplementary material for: Genome-scale data reveal the role of hybridization in lichen-forming fungi
Source: Sci Rep. 2020 Jan 30;10:1497. doi: 10.1038/s41598-020-58279-x (PMC6992703; doi:10.1038/s41598-020-58279-x)

Genome-scale data reveal the role of hybridization in lichen-forming fungi

**Supplementary Information**

Rachel Keuler^1^, Alexis Garretson^1^, Theresa Saunders^1^, Robert Erickson^1^, Nathan St. Andre^1^, Felix Grewe^2^, Hayden Smith^1^, H. Thorsten Lumbsch^2^, Jen-Pan Huang^3^, Larry L. St. Clair^1,4^, Steven D. Leavitt^1,4, *^

^1^*Department of Biology, Brigham Young University, 4102 Life Science Building, Provo, UT 84602, U.S.A.*

^2^*Grainger Bioinformatics Center, Science & Education, The Field Museum, 1400 S. Lake Shore Drive, Chicago, IL 60605, U.S.A.*

^3^*Biodiversity Research Center, Academia Sinica, 128 Academia Rd, Section 2, Nankang District, Taipei 11529, Taiwan*

^4^*M. L. Bean Life Science Museum, Brigham Young University, 1115 MLBM, Provo, UT 84602, U.S.A.*

^*^Corresponding author’s e-mail: steve_leavitt@byu.edu

**Figure S3**: Phylogenies inferred from mitochondrial phylogenomic datasets: Topologies were inferred using (a) concatenated CDS regions, (b) concatenated intronic regions, and (c) aligned complete draft mitochondrial genomes.


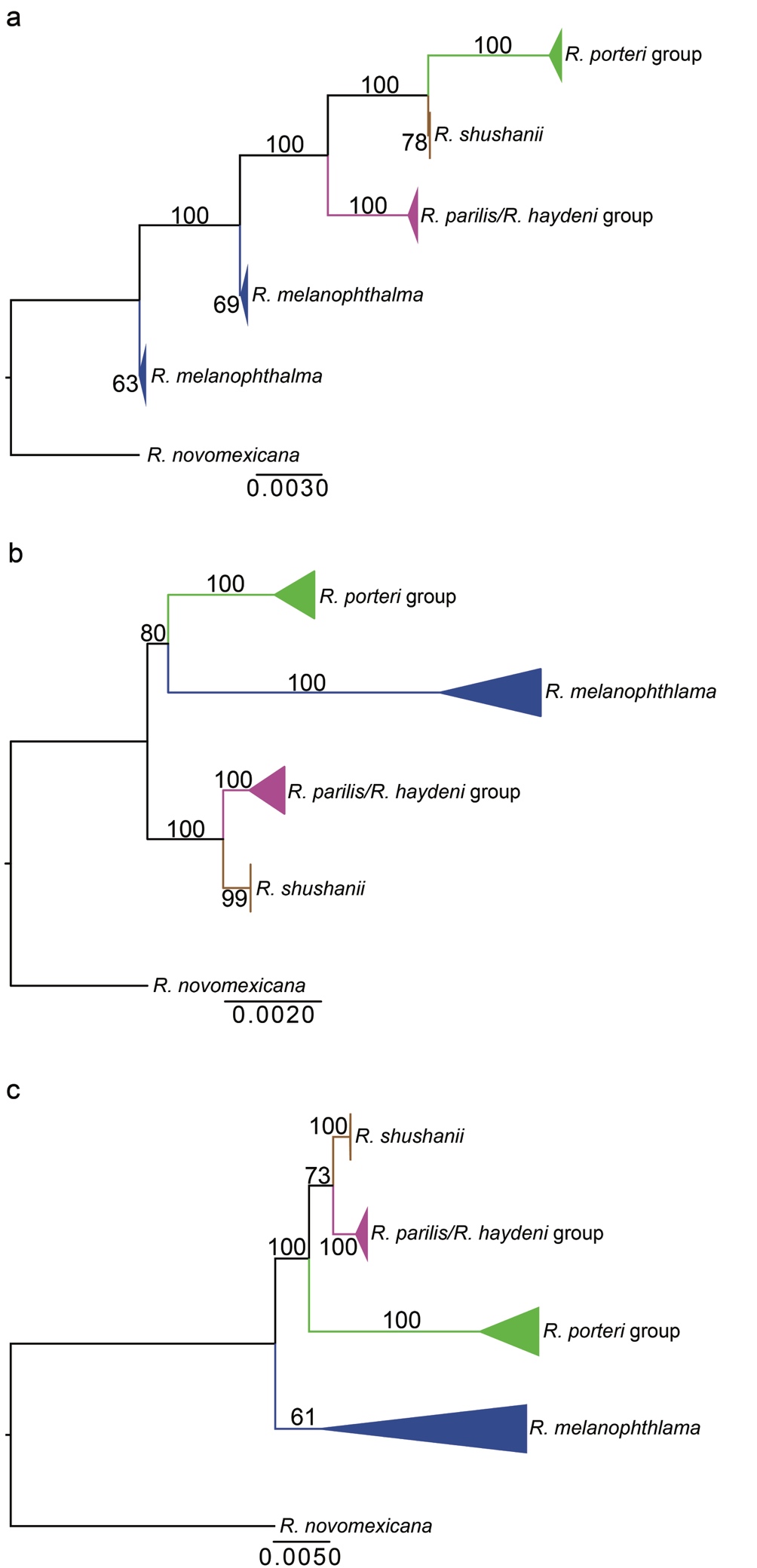

Supplement: Supplementary file 4 — Supporting Information 4. [file 41598_2020_58279_MOESM4_ESM.docx]
